# Network meta-analysis of the efficacy of different exercise therapies in patients with knee osteoarthritis

**DOI:** 10.7717/peerj.21900

**Published:** 2026-11-10

**Authors:** Kaiyue Diao, Tong Zhang, Hai Wang, Yu Wang, Yingxu Pan

**Affiliations:** 1 College of Physical Education and Training, Capital University of Physical Education and Sports, Beijing, China; 2 Physical Culture Institute, Weifang University, Weifang, Shandong, China; 3 China Volleyball Sports Institute, Beijing Sport University, Beijing, China

**Keywords:** Knee osteoarthritis, Exercise therapies, Efficacy, Network meta analysis

## Abstract

**Background:**

Knee osteoarthritis (KOA) is a leading cause of disability in the elderly and a growing social burden due to aging and increasing obesity rates. This study aims to investigate the effects of different exercise modalities on pain, physical function, stiffness, and muscle strength in patients with KOA.

**Methods:**

A systematic search was conducted in PubMed, Embase, Web of Science, Cochrane Library, and EBSCO databases for randomized controlled trials evaluating the efficacy of exercise therapy for KOA from January 2000 to 16 August 2025, followed by an updated search through 28 March 2026. No additional eligible studies were identified in the updated search. In total, 51 studies involving 3,045 participants (71.7% female; study-group mean ages ranged from 50.3 to 74.4 years) were included. The studies were conducted in 14 countries across Asia, Europe, North America, South America, and Oceania. A network meta-analysis was performed using Stata 16.0. This systematic review was registered with PROSPERO (CRD420251177632).

**Results:**

The results showed that mind-body exercise was the most effective in reducing pain (standardized mean differences (SMD) = −1.13, 95% confidence interval (CI) [−1.47 to −0.79], *P* < 0.001, Surface Under the Cumulative Ranking Curve (SUCRA) = 83%) and improving joint stiffness (SMD = −1.15, 95% CI [−1.59 to −0.70], *P* < 0.001, SUCRA = 78.5%). Mixed exercise ranked highest in improving knee function (SMD = −1.43, 95% CI [−2.31 to −0.55], *P* < 0.001, SUCRA = 81.8%), followed by mind-body exercise (SMD = −1.28, 95% CI [−1.64 to −0.93], *P* < 0.001, SUCRA = 77.4%). Strength training was most effective in enhancing muscle strength (SMD = 0.79, 95% CI [0.470–1.12], *P* < 0.001, SUCRA = 79.1%).

**Conclusion:**

Mind-body exercise appeared to be the most favorable option for reducing pain and stiffness in patients with knee osteoarthritis, whereas strength training appeared to be the most beneficial for improving muscle strength. These findings may help inform individualized exercise prescription, but should be interpreted cautiously given the uncertainty and limitations of the available evidence.

## Background

Osteoarthritis is one of the most common musculoskeletal disorders, affecting millions of people worldwide (Dell’Isola et al., 2025). Knee osteoarthritis (KOA) is a leading cause of disability in the elderly and a significant source of social costs. With the aging population and increasing obesity rates, this condition has become more prevalent than in previous decades (Glyn-Jones et al., 2015; Hunter & Bierma-Zeinstra, 2019).

Exercise is widely recognized as an effective conservative treatment for knee osteoarthritis. Numerous international clinical guidelines, such as those from the American College of Rheumatology (ACR) and the National Institute for Health and Care Excellence (NICE), recommend incorporating exercise as one of the standard treatment options for KOA (Holden et al., 2023; Kolasinski et al., 2020; Van Middelkoop et al., 2024). Existing systematic reviews and meta-analyses have demonstrated that exercise interventions, compared with non-exercise controls, not only reduce knee pain but also improve knee function, enhance patients’ quality of life, and are associated with fewer adverse effects (Fransen et al., 2015; Goff et al., 2021).

Exercise modalities differ in their structure and therapeutic emphasis. Aerobic exercise mainly involves continuous rhythmic activities aimed at improving cardiorespiratory endurance (Luo et al., 2025), whereas strength training uses external or bodyweight resistance to improve muscle strength and endurance (Ceballos-Laita et al., 2023). Mind-body exercise emphasizes coordinated movement, breathing regulation, postural control, and mind-body integration (Gao et al., 2025). Blood flow restriction training (BFRT) refers to low-load exercise performed with external blood flow restriction as a defining training feature and may provide an alternative for patients who have difficulty tolerating conventional high-load exercise (Wengle et al., 2022). In this review, mixed exercise (MEx) was defined as an intervention combining two or more co-primary exercise components when no single dominant component could be identified, such as aerobic exercise combined with strength training. When one exercise component was clearly predominant in the intervention structure or intended training effect, the intervention was classified according to that dominant component. These modalities represent structurally distinct exercise strategies and may produce different effects on pain, physical function, stiffness, and muscle strength.

Although the overall benefits of exercise for KOA are well established, uncertainty remains regarding the relative effectiveness of different exercise modalities, and the optimal modality may depend on the clinical outcome of interest. Therefore, this network meta-analysis aimed to compare mind–body exercise, aerobic exercise, strength training, BFRT, MEx, and non-exercise controls for reducing pain and stiffness and improving physical function and muscle strength in patients with KOA. By integrating direct and indirect evidence, this study sought to inform outcome-specific exercise selection and support more individualized exercise prescription.

## Method

This systematic review and network meta-analysis was conducted in accordance with the Preferred Reporting Items for Systematic Reviews and Meta-Analyses (PRISMA) guidelines (Hutton et al., 2015). The study protocol was prospectively registered in the International Prospective Register of Systematic Reviews (PROSPERO), with the registration number: CRD420251177632.

### Search strategy

We systematically searched PubMed, Embase, Cochrane Library, Web of Science, and EBSCO for studies published from January 2000 to 16 August 2025. To ensure that the review remained up to date, an updated search was subsequently conducted for studies published from 17 August 2025 to 28 March 2026. The search strategies were developed based on the key elements of the PICOS framework and combined controlled vocabulary terms with free-text terms. Specifically, Medical Subject Headings (MeSH) were used for PubMed and the Cochrane Library, and Emtree terms were used for Embase. The search strategies focused on three core concepts: knee osteoarthritis, exercise interventions, and randomized controlled trials. The detailed search strategies for all databases are provided in Appendix S2.

### Eligibility criteria

The inclusion criteria for this network meta-analysis were as follows: randomized controlled trials (RCTs), including individually randomized and cluster-randomized trials; participants diagnosed with knee osteoarthritis based on clinical or radiographic criteria; interventions involving exercise-based therapies; comparators including educational interventions, usual observation, or attention control; and studies reporting at least one of the following outcomes: pain, physical function, stiffness, or quadriceps muscle strength.

The exclusion criteria for this network meta-analysis were as follows: studies involving patients with knee osteoarthritis combined with other comorbidities (such as hypertension or diabetes); studies in which exercise interventions were combined with pharmacological treatments or surgical procedures; studies published as dissertations, conference proceedings, or abstracts; studies for which the full text was unavailable; duplicate publications; and review articles or meta-analyses.

### Study selection and data extraction

All records retrieved from the databases were imported into EndNote X9 for management and duplicate removal. Kaiyue Diao and Tong Zhang independently performed the literature search, study selection, and data extraction. Studies were first screened by title and abstract, followed by full-text assessment for eligibility. The extracted data included the first author, year of publication, country, sample size, participant characteristics (such as sex and age), intervention details, and outcome measures. Any disagreements were resolved through discussion or consultation with Yingxu Pan as the third reviewer. The updated screening process was completed following the literature search update on 28 March 2026, and no additional eligible studies were identified for inclusion.

### Exercise categories

Six categories were used to classify the exercise interventions in the included randomized controlled trials: control (CON), mind-body exercise (MB), strength training (ST), aerobic exercise (AE), BFRT, and mixed exercise (MEx). The detailed classification criteria are provided in Appendix S3.

### Risk of bias and GRADE assessment

The methodological quality of the included studies was independently assessed by two reviewers using the Cochrane Risk of Bias tool (Higgins et al., 2011). The overall risk of bias (ROB) for each study was evaluated as follows: a study was classified as having low ROB if none of the domains were rated as high risk and three or fewer domains were rated as unclear risk. A study was classified as having moderate ROB if one domain was rated as high risk or if no domains were rated as high risk but four or more domains were rated as unclear risk. All remaining studies were categorized as having high ROB (Cipriani et al., 2018). We used a GRADE-based framework adapted for network meta-analysis to assess the certainty of the evidence contributing to the network estimates for both primary and secondary outcomes. Following the established methodological framework (Salanti et al., 2014), the assessment considered study limitations, indirectness (including considerations of transitivity), inconsistency between direct and indirect evidence, imprecision, publication bias, and the contribution of individual comparisons to the network estimates.

### Statistical analysis

To minimize the impact of baseline differences, this study used change scores—defined as the mean and standard deviation differences from pre- to post-intervention—to pool effect sizes. Because the measurement units of the outcomes varied across studies, standardized mean differences (SMD) were used as the summary effect measure. All statistical analyses were performed using STATA version 16.0. A network evidence plot was generated to illustrate the relationships among the exercise interventions, in which each node represented a specific intervention, with the node size reflecting the total sample size, and the connecting lines indicating the presence of direct comparisons; the thickness of each line corresponded to the number of studies available for that direct comparison (Shim et al., 2017). Inconsistency within each closed loop of evidence was assessed using loop inconsistency tests, while global inconsistency was evaluated using the design-by-treatment interaction model. Local inconsistency between direct and indirect comparisons was examined using the node-splitting method. To compare and rank the relative effectiveness of different exercise modalities, we calculated the surface under the cumulative ranking curve (SUCRA) values to identify the most effective intervention (Salanti, Ades & Ioannidis, 2011). Publication bias or small-study effects were examined by visually inspecting funnel plots.

## Results

### Literature search results

The study selection process is shown in Fig. 1. A total of 11,189 records were initially identified, including 11,153 from database searches and 36 from reference lists. After duplicate removal, 7,204 records remained for title and abstract screening, of which 7,099 were excluded. The full texts of 105 articles were then assessed for eligibility, and 104 were excluded. Ultimately, 51 studies were included in the analysis. To update the search results, a supplementary search covering the period from 17 August 2025 to 28 March 2026 was conducted. After duplicate removal, 402 additional records remained for screening; however, no additional studies met the inclusion criteria. Therefore, the final number of included studies remained 51.

**Figure 1:**
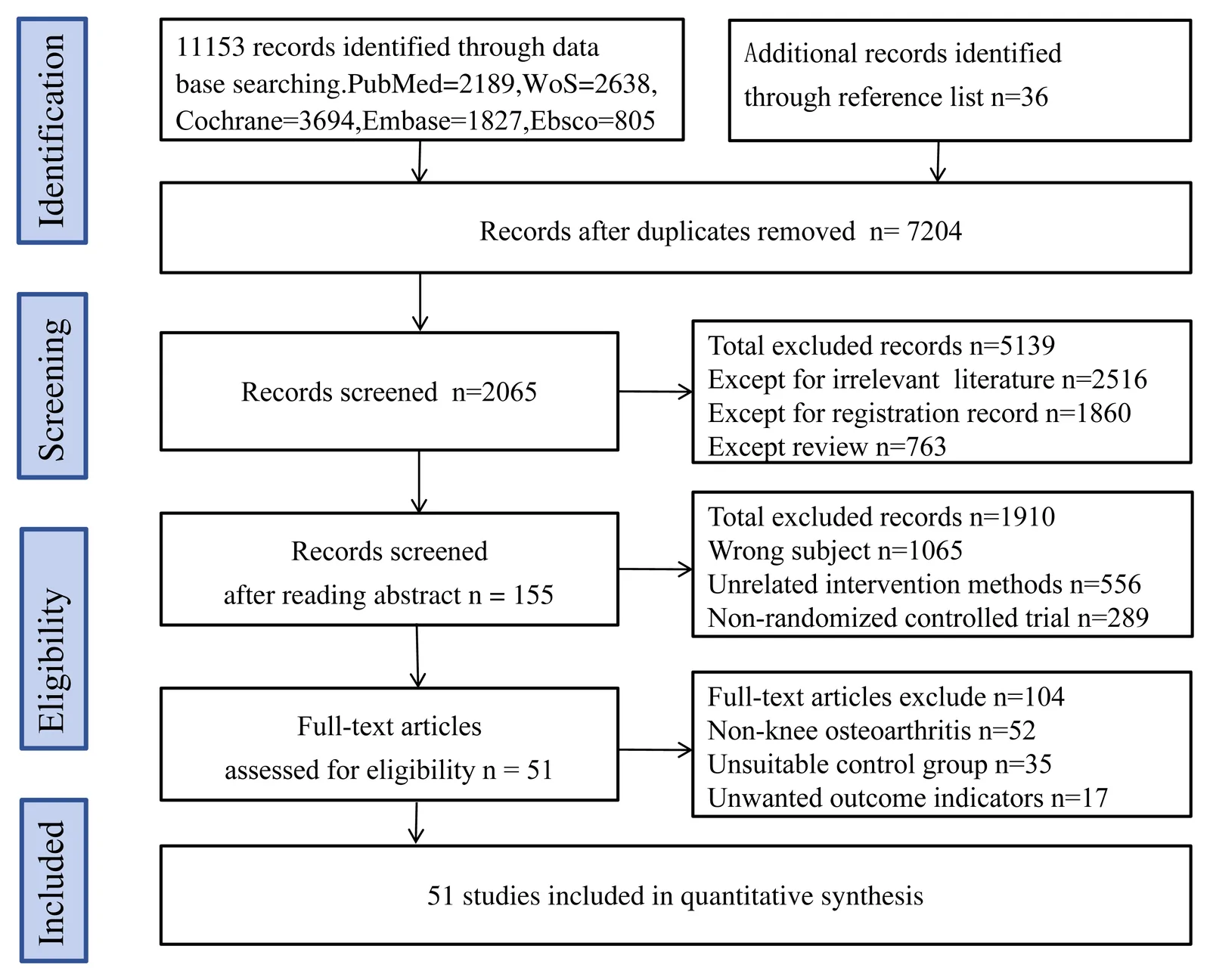
PRISMA flow diagram of the study selection process.

### Characteristics of the included studies

A total of 51 studies involving 3,045 participants were included, comprising 863 males and 2,182 females, with a mean age of 63.5 years. Among the exercise interventions, 1,880 participants were assigned to the experimental groups (MB: 550; ST: 752; AE: 379; BFRT: 95; MEx: 104), while 1,165 participants were allocated to the control groups. The intervention duration ranged from 4 to 48 weeks. All included studies reported at least one of the following outcomes: pain, physical function, stiffness, or muscle strength. Details are presented in Table 1.

**Table 1:** Basic characteristics of the included literature.

**Study (year)**	**Country**	**Participant**		**Intervention**			**Outcome**
		**Number T/C**	**Age T/C**	**T/C**	**Times/week**	**Week**	
Abafita et al. (2025)	Australia	59/58	63.7/61.2	ST/MB	3t/w	24w	WOMAC pain; function; Stiffness; Muscle strength;
Wortley et al. (2013)	United States	12/13/6	68.1/69.5/70.5	MB/ST/CON	2t/w	10w	WOMAC pain; function; Stiffness;
Wang et al. (2016)	United States	106/98	60.7/60.7	MB/CON	2t/w	12w	WOMAC pain; function; Stiffness;
Knoop et al. (2013)	United States	80/79	62.1/61.8	ST/CON	2t/w	12w	NRS; WOMAC function; Muscle strength;
Zhu et al. (2016)	CHINA	23/23	64.6/64.5	MB/CON	2t/w	24w	WOMAC pain; function; Stiffness;
Rabiei, Sheikhi & Letafatkar (2023)	Canada	27/27	59.8/61.2	MB/CON	3t/w	8w	WOMAC pain; function;
Henriksen et al. (2014)	Denmark	31/29	65.9/61.3	AE/CON	3t/w	12w	KOOS; KOOS function;
Sayers, Gibson & Cook (2012)	United States	12/11	66.9/68.4	ST/CON	3t/w	12w	WOMAC pain; function;
Wang et al. (2009)	United States	20/20	63.0/68.0	MB/CON	2t/w	12w	WOMAC pain; function; Stiffness;
Song et al. (2022)	CHINA	20/20	64.2/64.2	MB/CON	3t/w	12w	WOMAC pain; function; Stiffness;
Branco Ferraz et al. (2018)	Brazil	16/16	59.9/60.3	ST/BFRT	2t/w	12w	WOMAC pain; function; Stiffness; Muscle strength;
Bryk et al. (2016)	Brazil	17/17	62.3/60.4	BFRT/CON	3t/w	6w	NPRS; Quadriceps strength;
Lin et al. (2009)	CHINA	36/36	62.2/61.6	ST/CON	3t/w	8w	WOMAC pain; function; Muscle strength;
Salacinski et al. (2012)	United States	19/18	55.1/60.6	AE/CON	2t/w	12w	WOMAC pain; function; Stiffness; Muscle strength;
Yuenyongviwat et al. (2020)	Thailand	42/44	62.8/62.5	ST/CON	3t/w	10w	KOOS;
Kang et al. (2022)	CHINA	12/15	63.4/64.7	MB/CON	3t/w	36w	VAS; WOMAC function; Stiffness;
Kuntz et al. (2018)	Canada	10/11/10	65.5/65.5/71.1	MB/ST/CON	3t/w	12w	KOOS; KOOS function; Stiffness; Muscle strength;
Thorstensson et al. (2005)	Sweden	30/31	54.8/57.3	ST/CON	2t/w	6w	KOOS;
Drummen et al. (2023)	Australia	24/24	65.1/67.4	AE/CON	3t/w	24w	WOMAC pain; function; Stiffness; Muscle strength;
Alghadir et al. (2019)	Saudi Arabia	23/22	54.6/56.8	AE/CON	3t/w	6w	NRS; WOMAC function; Muscle strength;
Oiestad et al. (2023)	Norway	53/54/54	57.3/57.6/57.8	ST/AE/CON	3t/w	14w	KOOS; Muscle strength;
Wang et al. (2021)	CHINA	41/43/44	64.7/65.7/65.5	ST/MB/MEx	3t/w	24w	WOMAC pain; function;
Cheung et al. (2017)	United States	32/28/23	68.9/74.4/71.8	MB/MEx/CON	4t/w	8w	WOMAC pain; function; Stiffness;
Jorge et al. (2015)	Brazil	29/31	61.7/59.9	ST/CON	2t/w	12w	WOMAC pain; function; Stiffness; Muscle strength;
Zhang et al. (2021)	CHINA	25/25	53.4/55.7	MB/ST	2t/w	12w	WOMAC pain; function; Stiffness;
Ye et al. (2020)	CHINA	25/25	64.5/63.1	MB/CON	3t/w	12w	WOMAC pain; function; Stiffness;
Mazloum et al. (2018)	Iran	14/14/13	50.3/55.0/50.8	MB/ST/CON	3t/w	8w	Lequesne index
Harper et al. (2019)	United States	19/16	69.1/67.2	ST/BFRT	3t/w	12w	WOMAC pain
Hiyama et al. (2012)	Japan	20/20	71.9/73.8	AE/CON	-	4w	JKOM
Jan et al. (2008)	CHINA	34/30	63.3/62.8	ST/CON	3t/w	8w	WOMAC pain; WOMAC function; Muscle strength;
Pan et al. (2025)	CHINA	12/12	65.2/62.9	MB/CON	3t/w	14w	WOMAC pain; function; Stiffness; Muscle strength;
Cheung et al. (2014)	United States	18/18	71.9/71.9	MB/CON	4t/w	8w	WOMAC pain; function; Stiffness;
Rego et al. (2023)	Brazil	8/9	65.7/64.8	MB/CON	2t/w	7w	WOMAC pain; function; Stiffness;
Ye et al. (2019)	CHINA	28/28	65.1/63.6	MB/CON	3t/w	12w	WOMAC pain; function; Stiffness;
Gomiero et al. (2018)	Brazil	32/32	61.8/61.6	ST/MEx	2t/w	16w	VAS;
Ni et al. (2010)	CHINA	14/15	62.9/63.5	MB/CON	2-4t/w	24w	WOMAC pain; function; Stiffness;
Zhu et al. (2017)	CHINA	23/23	64.6/64.5	MB/CON	3t/w	24w	WOMAC pain; Stiffness; Muscle strength;
Segal et al. (2015)	United States	19/21	56.1/54.6	BFRT/CON	3t/w	4w	KOOS; Muscle strength;
Grossl et al. (2023)	Brazil	13/13	63.3/65.5	ST/BFRT	2t/w	12w	VAS; Muscle strength;
Chen et al. (2021b)	CHINA	16/16	60.3/60.9	AE/CON	3t/w	4w	WOMAC pain; function; Stiffness;
Brosseau et al. (2012)	Canada	43/41	63.9/62.3	AE/CON	3t/w	48w	WOMAC pain; function; Stiffness;
Chen et al. (2019)	CHINA	71/70	68.9/68.8	ST/CON	3t/w	12w	WOMAC pain; Stiffness;
Waller et al. (2017)	Australia	43/44	63.8/63.9	ST/CON	3t/w	16w	KOOS;
Wallis et al. (2017)	Australia	23/23	68.0/67.0	AE/CON	_	12w	WOMAC pain; Stiffness;
Ha et al. (2018)	Korea	9/8	60.9/61.3	AE/CON	3t/w	12w	WOMAC pain; function; Stiffness;
Dias et al. (2017)	Brazil	33/32	70.8/71.0	AE/CON	2t/w	6w	WOMAC pain; WOMAC function; Muscle strength;
Sari et al. (2023)	India	14/14	61.4/57.7	ST/BFRT	2t/w	6w	WOMAC pain; function; Stiffness;
Foley et al. (2003)	Australia	32/35/35	69.8/73.0/69.8	ST/AE/CON	3t/w	6w	WOMAC pain; function; Stiffness;
Bokaeian et al. (2021)	Iran	20/18/18	54.9/57.0/56.7	MB/ST/AE	3t/w	4w	VAS; Muscle strength;
DeVita et al. (2018)	United States	15/15	58.1/56.2	ST/CON	3t/w	12w	WOMAC pain; function; Muscle strength;
Taglietti et al. (2018)	Brazil	31/29	67.3/68.7	AE/CON	2t/w	8w	WOMAC pain

**Notes.**

Abbreviations T/C treatment/control group CON control group ST strength training MB mind-body exercise AE aerobic exercise BFRT blood flow restriction training MEx mixed exercise WOMAC Western Ontario and McMaster Universities Osteoarthritis Index NRS Numeric Rating Scale NPRS Numeric Pain Rating Scale KOOS Knee injury and Osteoarthritis Outcome Score VAS Visual Analog Scale JKOM Japanese Knee Osteoarthritis Measure t/w times per week w weeks

Pain and stiffness were assessed using validated patient-reported measures. Pain was commonly evaluated using the WOMAC pain subscale, Visual Analog Scale (VAS), Numeric Rating Scale (NRS)/Numeric Pain Rating Scale (NPRS), and other knee-specific measures such as the Knee injury and Osteoarthritis Outcome Score (KOOS), whereas stiffness was predominantly assessed using the WOMAC stiffness subscale. Muscle strength was assessed using objective strength-testing methods, including isokinetic measurements of quadriceps or knee-extensor peak torque, isometric quadriceps strength testing, and dynamic strength assessments such as one-repetition maximum (1RM) knee-extension or leg-press tests.

### Risk of bias assessment

Details of the risk-of-bias assessment for each study are provided in Appendix S4. A total of 40 studies clearly reported methods of random sequence generation, 38 reported allocation concealment, and 36 described blinding of outcome assessment. For incomplete outcome data, studies with loss to follow-up greater than 20% without intention-to-treat analysis were considered at high risk. Among the included studies, 6 had loss to follow-up exceeding 20%, whereas 45 studies reported attrition with similar numbers and reasons for dropout between intervention and control groups.

Additional study characteristics, including very small sample size, supervision status of the exercise intervention, and reported conflicts of interest, were extracted and considered during interpretation; however, these factors were not uniformly treated as automatic indicators of high risk under the “other bias” domain without further methodological justification. Overall, 41 studies were assessed as having low risk of bias, eight as moderate risk, and two as high risk.

### Network meta-analysis results

#### Network evidence map

Figure 2 presents the network evidence map illustrating the comparative effects of different exercise modalities for patients with knee osteoarthritis. Strength training (ST) was the most frequently studied intervention, followed by mind-body exercise (MB) and aerobic exercise (AE). All intervention pairs had mixed evidence except for AE *vs.* BFRT, AE *vs.* MEx, and BFRT *vs.* MEx, which were supported solely by indirect comparisons. Appendix S5 provides the contribution matrix of direct and indirect comparisons to the network meta analysis (NMA) estimates, as well as the number of studies included in each direct comparison.

**Figure 2:**
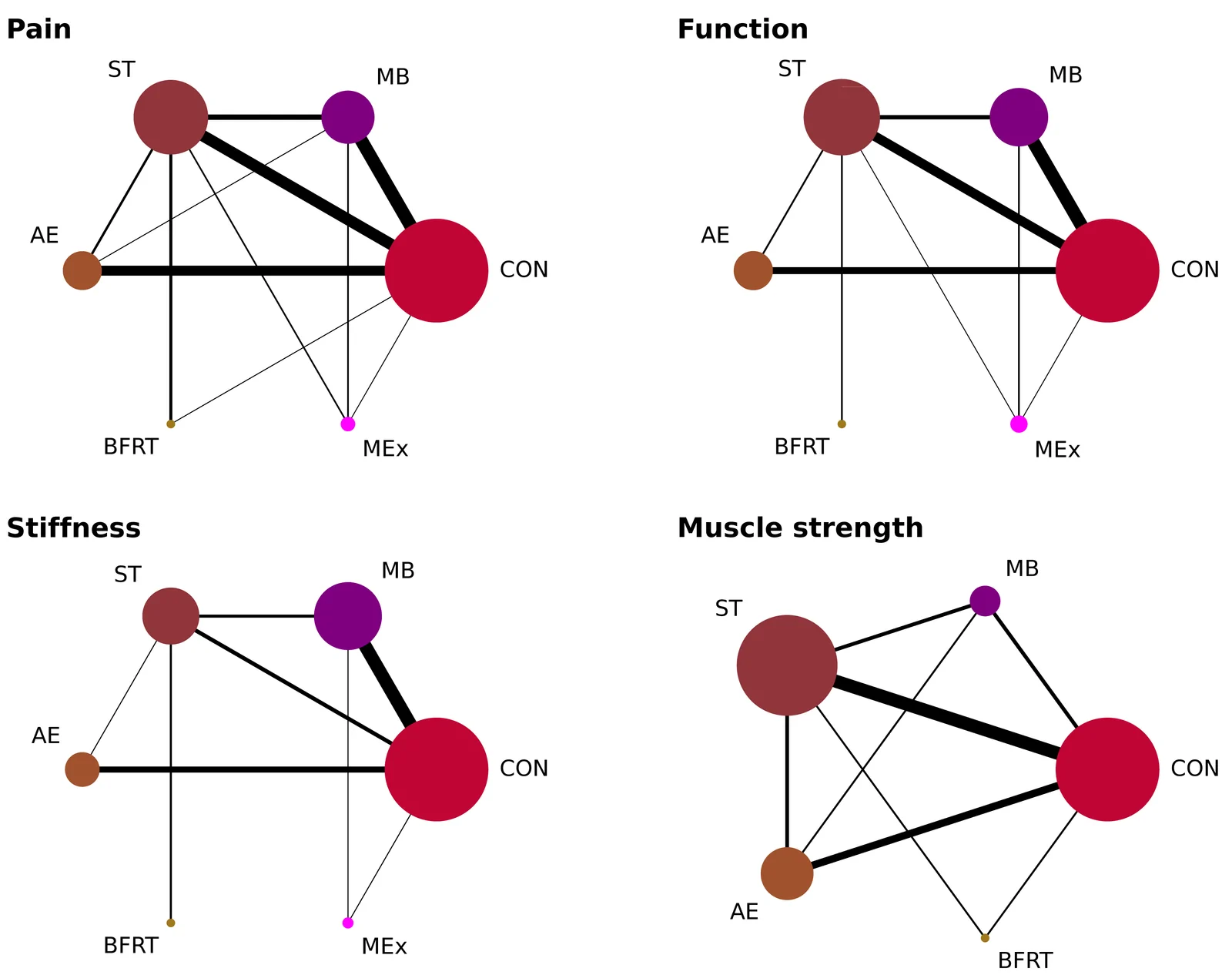
Network plots of exercise interventions for pain, function, stiffness, and muscle strength. Each node represents an intervention, and the node size is proportional to the total number of participants assigned to that intervention. A line between two nodes indicates a direct comparison, and the line thickness is proportional to the number of studies contributing to that direct comparison. CON, control; MB, mind–body exercise; ST, strength training; AE, aerobic exercise; BFRT, blood flow restriction training; MEx, mixed exercise.

#### Inconsistency assessment

Inconsistency across the network was assessed using loop inconsistency tests, the design-by-treatment interaction model, and the node-splitting method (Appendix S6). The loop inconsistency tests indicated no statistically significant inconsistency in any triangular loop for pain, physical function, or stiffness (all *P* > 0.05). For muscle strength, inconsistency was detected only in the CON–MB–AE loop; however, after exclusion of Bokaeian et al. (2021), this loop was no longer statistically inconsistent. The intervention in this study combined yoga with medial-thrust gait training and was delivered over a relatively short 4-week period. These differences in intervention composition and duration may represent potential effect modifiers and may have reduced transitivity within the loop. Nevertheless, the exclusion analysis identifies an influential study rather than establishing a definitive causal explanation for the inconsistency. The design-by-treatment interaction model showed no significant global inconsistency for any outcome (all *P* > 0.05), suggesting that a consistency model was appropriate for the analyses. The node-splitting results also showed no statistically significant differences between direct and indirect evidence across outcomes (all *P* > 0.05), indicating no important local inconsistency.

Forest plots for eligible comparisons of pain, physical function, stiffness, and muscle strength, including 95% confidence intervals and 95% prediction intervals, are provided in Appendix S7.

### Pooled effect estimates and ranking results

For the pain outcome, all exercise modalities (MB, ST, AE, BFRT, and MEx) were associated with significant reductions compared with the no-exercise control group (Table 2). Specifically, significant effects were observed for MB (SMD = −1.13; 95% CI [−1.47 to −0.79]; *P* < 0.001), ST (SMD = −0.68; 95% CI [−1.01 to −0.35]; *P* < 0.01), AE (SMD = −0.87; 95% CI [−1.26 to −0.48]; *P* < 0.001), BFRT (SMD = −0.93; 95% CI [−1.66 to −0.19]; *P* = 0.014), and MEx (SMD = −1.01; 95% CI [−1.79 to −0.22]; *P* = 0.012). Among the active interventions, MB produced a significantly greater reduction in pain than ST. According to the SUCRA results (Table 3), MB ranked highest (83.0%), followed by MEx (66.9%), BFRT (60.6%), AE (55.6%), and ST (33.6%). The corresponding cumulative ranking probability curves for all outcomes are shown in Fig. 3. These ranking results were broadly consistent with the pooled effect estimates but should be interpreted alongside the uncertainty reflected in the confidence intervals.

For the function outcome, all exercise modalities showed significant improvements compared with the no-exercise control group, including MB (SMD = −1.28; 95% CI [−1.64 to −0.93]; *P* < 0.001), ST (SMD = −0.91; 95% CI [−1.29 to −0.53]; *P* < 0.001), AE (SMD = −0.87; 95% CI [−1.36 to −0.38]; *P* < 0.001), and MEx (SMD = −1.43; 95% CI [−2.31 to −0.55]; *P* < 0.001). In the ranking analysis, MEx ranked highest (81.8%), followed by MB (77.4%), BFRT (57.5%), ST (42.0%), and AE (40.8%). Although MEx and MB ranked more favorably, interpretation should consider the width of the confidence intervals and the uncertainty of the comparative estimates.

**Table 2:** Network meta-analysis matrix of outcomes.

**Pain**	MB					
	**−0.45 (−0.85, −0.05)**	ST				
	−0.26 (−0.76, 0.24)	0.19 (−0.29, 0.67)	AE			
	−0.20 (−0.98, 0.58)	0.25 (−0.45, 0.94)	0.06 (−0.76, 0.87)	BFRT		
	−0.12 (−0.91 ,0.66)	0.32 (−0.46, 1.10)	0.13 (−0.73, 1.00)	0.08 (−0.95, 1.11)	MEx	
	**−1.13 (−1.47, −0.79)**	**−0.68 (−1.01, −0.35)**	**−0.87 (−1.26, −0.48)**	**−0.93 (−1.66, −0.19)**	**−1.01 (−1.79, −0.22)**	CON
**Function**	MB					
	−0.37 (−0.82, 0.07)	ST				
	−0.42 (−1.02, 0.18)	−0.04 (−0.62, 0.53)	AE			
	−0.21 (−1.35, 0.92)	0.16 (−0.89, 1.21)	0.20 (−0.99, 1.40)	BFRT		
	0.14 (−0.72, 1.01)	0.52 (−0.38, 1.41)	0.56 (−0.43, 1.56)	0.36 (−1.02, 1.74)	MEx	
	**−1.28 (−1.64, −0.93)**	**−0.91 (−1.29, −0.53)**	**−0.87 (−1.36, −0.38)**	−1.07 (−2.18, 0.04)	**−1.43 (−2.31, −0.55)**	CON
**Stiffness**	MB					
	−0.35 (−1.03, 0.32)	ST				
	−0.51 (−1.29, 0.26)	−0.16 (−1.03, 0.70)	AE			
	0.01 (−1.34, 1.36)	0.36 (−0.81, 1.53)	0.52 (−0.93, 1.98)	BFRT		
	−0.35 (−1.73, 1.03)	0.00 (−1.50, 1.50)	0.17 (−1.36, 1.69)	−0.36 (−2.26, 1.55)	MEx	**–**
	**−1.15 (−1.59, −0.70)**	**−0.79 (−1.44, −0.15)**	−0.63 (−1.28, 0.02)	−1.15 (−2.49, 0.19)	−0.80 (−2.18, 0.59)	CON
**Muscle strength**	MB					
	−0.29 (−0.80, 0.22)	ST				
	−0.13 (−0.72, 0.47)	0.16 (−0.31, 0.63)	AE			
	−0.23 (−1.13, 0.66)	0.06 (−0.71, 0.82)	−0.10 (−0.96, 0.75)	BFRT	–	
	–	–	–	–	MEx	**–**
	0.50 (−0.01, 1.02)	**0.79 (0.47, 1.12)**	**0.63 (0.22, 1.05)**	0.74 (−0.03, 1.51)	–	CON

**Notes.**

League table values are expressed as effect sizes (95% confidence intervals) for pairwise comparisons between the column and row interventions (column minus row). For pain, function, and stiffness, negative values indicate that the column intervention is favored, whereas positive values indicate that the row intervention is favored. For muscle strength, positive values indicate that the column intervention is favored, whereas negative values indicate that the row intervention is favored. Bold type indicates statistical significance.

**Table 3:** SUCRA rankings.

Pain	SUCRA(%)	Function	SUCRA(%)	Stiffness	SUCRA(%)	Muscle Strength	SUCRA(%)
MB	83	MEx	81.8	MB	78.5	ST	79.1
MEx	66.9	MB	77.4	BFRT	71.8	BFRT	67.6
BFRT	60.6	BFRT	57.5	MEx	53	AE	58.1
AE	55.6	ST	42	ST	51.2	MB	43.8
ST	33.6	AE	40.8	AE	41.3	CON	1.3
CON	0.3	CON	0.6	CON	4.2	–	–

**Figure 3:**
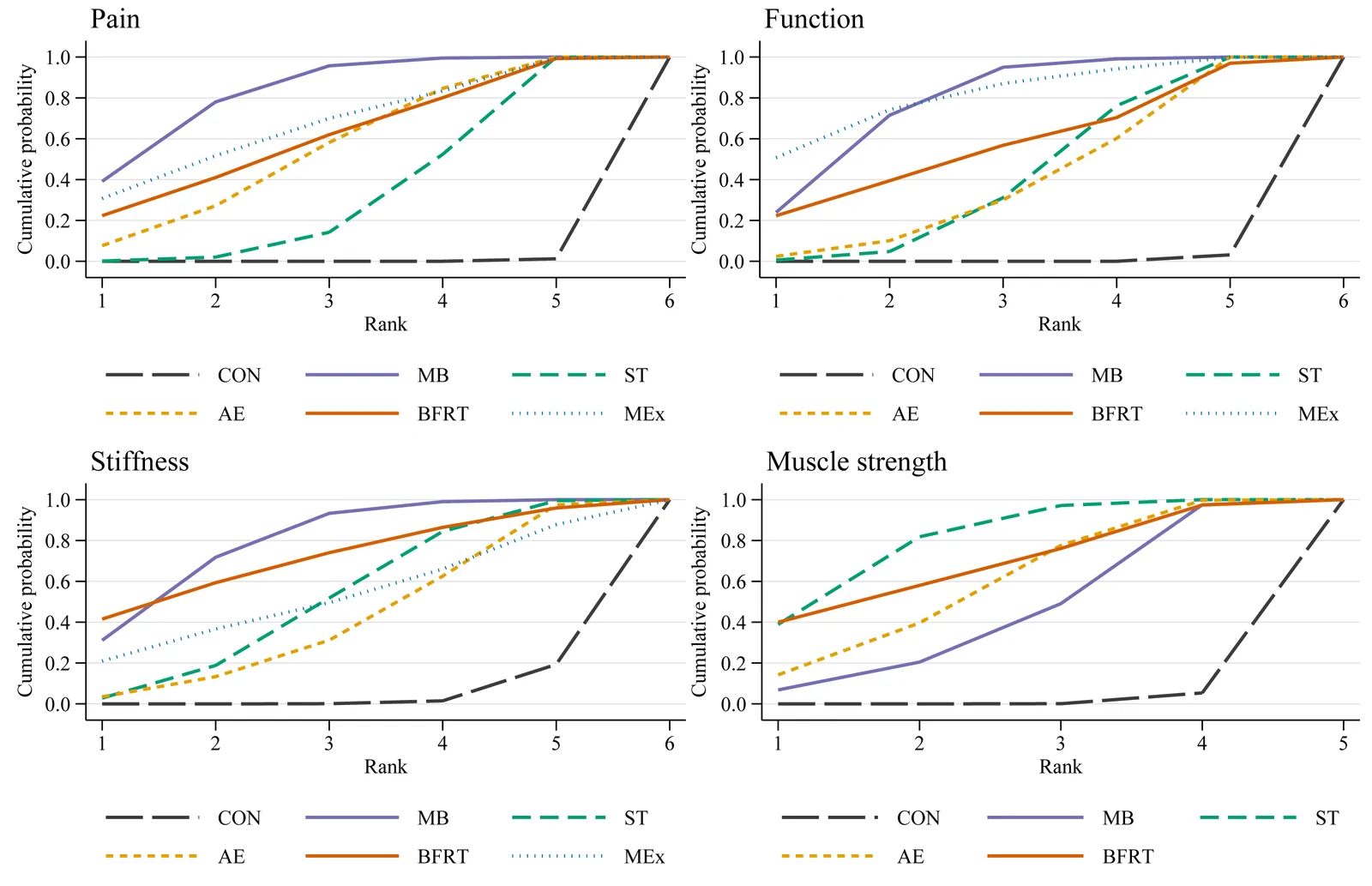
SUCRA ranking curves of exercise interventions.

For the stiffness outcome, MB and ST showed significant reductions compared with the no-exercise control group. Specifically, MB (SMD = −1.15; 95% CI [−1.59 to −0.70]; *P* < 0.001) and ST (SMD = −0.79; 95% CI [−1.44 to −0.15]; *P* < 0.01) demonstrated statistically significant improvements. In the ranking analysis, MB ranked highest (78.5%), whereas ST ranked fourth (51.2%). These findings suggest that MB may be among the more favorable interventions for stiffness reduction, although the ranking results should be interpreted cautiously.

For the muscle strength outcome, significant improvements compared with the no-exercise control group were observed for ST (SMD = 0.79; 95% CI [0.47–1.12]; *P* < 0.001) and AE (SMD = 0.63; 95% CI [0.22–1.05]; *P* < 0.01), whereas MB and BFRT did not show statistically significant differences from the control group. No effect estimate was available for MEx because none of the included studies involving MEx reported muscle strength outcomes eligible for inclusion in the present analysis. Accordingly, MEx was not included in the SUCRA ranking analysis for muscle strength. Among the interventions with available data, ST ranked highest (79.1%), whereas AE ranked third (58.1%). Therefore, the ranking results for muscle strength were aligned with the pooled estimates in indicating that ST was the most favorable intervention for this outcome, while neither MB nor BFRT showed a statistically significant advantage over the control group.

### Publication bias

Publication bias was assessed using funnel plots for each outcome measure. The funnel plots showed good symmetry, suggesting minimal publication bias or small-study effects. The results are presented in Appendix S8.

### Restricted analyses of primary outcomes

Restricted analyses of the primary outcomes were conducted according to participant age and intervention duration to explore whether these study-level characteristics influenced the results. For age, analyses were restricted to studies involving participants aged 50–60 years. For intervention duration, analyses were repeated after excluding short-term interventions (4–7 weeks), thereby retaining only medium- and long-term interventions. Overall, these restricted analyses did not materially alter the main findings, suggesting that the results were generally stable. Detailed league tables are presented in Appendix S9, and SUCRA ranking plots for the restricted analyses are shown in Appendix 10.

### GRADE assessment

Appendix S11 presents the GRADE assessment results for each comparison. Overall, the certainty of evidence for most comparisons was rated as moderate to low for pain and muscle strength outcomes, whereas most comparisons for knee function and stiffness were rated as low to very low.

## Discussion

Although numerous systematic reviews and meta-analyses have established the positive clinical outcomes of various exercises in the treatment of KOA, consensus on the optimal exercise modality remains difficult to reach. This study utilized network meta-analysis to compare the efficacy of MB, ST, AE, BFRT, and MEx for KOA. Our findings suggest that MB appeared to be among the more favorable interventions for reducing pain and stiffness. MEx ranked highest for improving knee function, followed by MB, although these findings should be interpreted in light of the uncertainty of the comparative estimates. ST appeared to be the most favorable intervention for improving muscle strength.

Previously, Uthman et al. (2013) conducted a network meta-analysis and accumulated sufficient evidence indicating that exercise provides significant benefits for patients with osteoarthritis compared to no exercise. Subsequently, Goh et al. (2019) introduced new outcome measures (performance and quality of life) and concluded that the effects of exercise vary depending on the type of exercise and the targeted outcome. Aerobic exercise or mind-body exercise may be the most effective in improving pain and function, which is consistent with the findings of our study. However, the subjects in both studies included patients with both knee and hip osteoarthritis, which may have influenced the final results due to the heterogeneity of the study population. In contrast, our analysis only included patients with knee osteoarthritis and no other comorbidities, which enhanced the specificity and made our results more reliable. Additionally, our study interventions did not involve neurostimulation, electrotherapy, or other combined treatments with exercise, allowing for a more accurate investigation of the effects of different types of exercise on patients with knee osteoarthritis.

The results of this study indicate that mind-body exercises (such as Tai Chi, yoga, Pilates, *etc*.) can reduce pain and improve stiffness in patients with knee osteoarthritis, while enhancing overall health through meditation and breathing exercises (Bennell et al., 2022; Wen et al., 2022). These types of exercises are particularly suitable for patients with chronic pain, especially among the elderly population (Wang et al., 2016). For example, Tai Chi, a traditional Chinese mind-body exercise that combines slow movements, breath control, and mindfulness, helps improve functional mobility and alleviate pain symptoms in elderly individuals with knee osteoarthritis, and is highly safe (Chen et al., 2021a; Kang et al., 2022; Ni et al., 2010; Zhu et al., 2016). Yoga, which incorporates body postures, breathing techniques, and meditation or relaxation, has been suggested by Abafita, Cheung, and others as a potential alternative or complementary exercise in clinical practice to help manage knee osteoarthritis, showing significant effects in reducing pain (Abafita et al., 2025; Cheung et al., 2017; Cheung et al., 2014; Kuntz et al., 2018). Rego, Mazloum, and others believe that Pilates can improve the health, pain, and function of elderly women with knee osteoarthritis (Mazloum et al., 2018; Rego et al., 2023).

Mixed exercise refers to when two or more types of exercises are combined, such as aerobic exercise combined with strength training or mind-body exercise combined with strength training. We found that mixed exercise has a positive effect on improving knee joint function (Gomiero et al., 2018), which is in contrast to the results concluded by Goh et al. (2019). Wang et al. (2021) suggested that Baduanjin combined with quadriceps strength training is more effective than either Baduanjin or quadriceps strength training alone in enhancing physical function, improving self-efficacy, and increasing quality of life. Cheung et al. (2017) believed that both yoga combined with aerobic exercise and strength training (ASE) could improve knee joint function, although three non-serious adverse events were reported in the ASE group. However, due to the insufficient overall literature on mixed exercise, further research evidence is needed.

Studies have found that strength training is most effective in improving quadriceps strength in the knee joint. Several studies have verified the important role of strength training in alleviating pain, enhancing muscle strength, and reducing joint damage in patients with knee osteoarthritis (Jorge et al., 2015; Wortley et al., 2013). By increasing the strength of the knee muscles, patients are better able to support the knee joint, reducing joint instability and pain caused by muscle weakness (Li et al., 2016). Additionally, some studies suggest that strength training can significantly improve gait speed in patients with knee osteoarthritis (Li et al., 2021).

Some studies suggest that in patients with knee osteoarthritis, aerobic exercise may be the most beneficial type of exercise for improving pain, function, gait performance, and quality of life, with moderate certainty (Yan et al., 2025). However, in this study, we found that although aerobic exercise has advantages in alleviating pain, enhancing muscle function, and increasing muscle strength in patients with knee osteoarthritis, it is not the best type of exercise. More research and long-term follow-up are needed to provide further evidence.

BFRT, as a relatively new treatment modality, did not show a clear advantage in the present analysis. However, it offers a low-load, low-risk training option for patients with knee osteoarthritis, particularly those who are unable to engage in high-intensity training (Wengle et al., 2022; Whiteley, 2019). BFRT has similar effects to strength training in increasing the maximum dynamic strength of the lower limbs in patients with knee osteoarthritis (Baker et al., 2020). Furthermore, compared to resistance exercise alone, incorporating BFRT into a traditional exercise regimen can significantly improve both short-term and long-term outcomes in patients with knee osteoarthritis, showing sustained improvements in pain, symptoms, and functional measures (Gopinatth et al., 2025; Jacobs et al., 2024).

Beyond differences in exercise modality, intervention duration varied considerably across studies, ranging from 4 to 48 weeks, and may have contributed to clinical heterogeneity. In the restricted analysis of the primary outcomes, exclusion of short-term interventions lasting 4–7 weeks yielded results that were broadly consistent with the main analysis, suggesting that the principal findings were relatively robust to the exclusion of the shortest interventions. Nevertheless, intervention duration varied alongside other features of the exercise programs, including frequency, intensity, and program design, making it difficult to isolate the effect of duration itself. Further studies are needed to clarify the role of intervention duration and to determine whether an optimal treatment period can be identified.

These findings may help inform individualized exercise prescription for patients with knee osteoarthritis. For patients whose primary treatment goal is pain relief, mind-body exercise may be considered a favorable option. For those seeking improvement in knee function, mixed exercise may represent a promising approach, although the heterogeneity within this category should be taken into account. For patients with reduced muscle strength, strength training appeared to be the most favorable intervention for improving muscle strength, while aerobic exercise may also provide benefit as a comprehensive form of exercise. In addition, blood flow restriction training may represent a potentially useful low-load option for patients who are unable to tolerate high-intensity training; however, further research is needed to confirm its long-term effects. Overall, these findings may support clinical decision-making, but they should be interpreted together with effect sizes, uncertainty, and the certainty of the available evidence.

### Study limitations

This study has several limitations. The included interventions varied substantially in exercise intensity, frequency, duration, and program design, which may have introduced clinical heterogeneity. In addition, most studies had short follow-up periods or lacked follow-up data, limiting the assessment of long-term effects. Women accounted for 71.7% of the included participants, resulting in a substantial sex imbalance. Given reported sex-related differences in the prevalence and clinical manifestations of knee osteoarthritis, the generalisability of the present findings to male populations may therefore be limited. Future studies with more balanced sex representation and sex-stratified analyses are warranted. Several outcomes, including pain, stiffness, and physical function, were assessed using self-reported measures such as the WOMAC, KOOS, and VAS, which may be susceptible to response bias, including social desirability bias. In addition, the included studies were conducted across multiple countries and cultural settings, and differences in language and cultural interpretation of questionnaire items may have introduced measurement heterogeneity and affected cross-study comparability. The mixed exercise category may also contain heterogeneous combinations of exercise components, and none of the included studies involving MEx reported muscle strength outcomes eligible for inclusion in the present analysis. Therefore, the relative effectiveness of MEx for improving muscle strength could not be estimated, which limits the comprehensiveness of the evidence for this outcome. Some potential effect modifiers were also not consistently reported across studies. Furthermore, although a GRADE-based approach adapted for network meta-analysis was used, CINeMA was not applied; therefore, this should be considered a methodological limitation of the study. Future research is needed to clarify the influence of specific exercise variables and to examine the long-term effects of these therapies in patients with knee osteoarthritis.

## Conclusion

Based on the available evidence, mind-body exercise appeared to be the most favorable intervention for reducing pain and stiffness in patients with knee osteoarthritis, whereas strength training appeared to be the most beneficial for improving muscle strength. These findings may help inform individualized exercise prescription, but should be interpreted cautiously in light of the uncertainty and limitations of the available evidence.

## Supplemental Information

10.7717/peerj.21900/supp-1 Supplemental Information 1 PRISMA Checklist

10.7717/peerj.21900/supp-2 Supplemental Information 2 Supplemental figures, tables, search strategies, and intended audience information

## Additional Information and Declarations

### Data Availability

The following information was supplied regarding data availability: This is a Systematic review/Meta-analysis.
