# Supplementary material for: Network meta-analysis of the efficacy of different exercise therapies in patients with knee osteoarthritis
Source: PeerJ. 2026 Nov 10;14:e21900. doi: 10.7717/peerj.21900 (PMC13762337; doi:10.7717/peerj.21900)
Supplement: Supplementary file 2 [file peerj-14-21900-s002.docx]

**Appendix 1**

**Intended Audience**

This study is intended for professionals involved in the diagnosis, treatment, and rehabilitation management of knee osteoarthritis, including orthopedic clinicians, rehabilitation physicians, physical therapists, sports rehabilitation practitioners, and researchers in sports medicine. The findings may also be relevant to clinical practice guideline developers and health decision-makers.

Exercise interventions for knee osteoarthritis are diverse, and understanding the relative efficacy and clinical applicability of different exercise therapies is important for treatment selection in both clinical and rehabilitation settings. By synthesizing the available evidence through a systematic review and meta-analysis, this study aims to support evidence-based exercise prescription for knee osteoarthritis, assist professionals in optimizing intervention decisions, and inform future research, evidence updates, and guideline development.

**Appendix 2**.

**Search strategy of Cochrane**

#1 (Knee Osteoarthritis):ti,ab,kw or (Osteoarthritides):ti,ab,kw or (Osteoarthrosis):ti,ab,kw or (Osteoarthroses):ti,ab,kw or (Arthritis, Degenerative):ti,ab,kw or (Arthritides, Degenerative):ti,ab,kw or (Degenerative Arthritides):ti,ab,kw or (Degenerative Arthritis):ti,ab,kw or (Arthrosis):ti,ab,kw or (Arthroses):ti,ab,kw or (Osteoarthrosis Deformans):ti,ab,kw

#2 (Aerobics):ti,ab,kw or (Physical Exercise):ti,ab,kw or (Aerobic Exercise):ti,ab,kw or (walk):ti,ab,kw or (run):ti,ab,kw or (dance):ti,ab,kw or (swim):ti,ab,kw or (aquatic exercises):ti,ab,kw or (bike):ti,ab,kw or (bicycle):ti,ab,kw or (treadmill):ti,ab,kw or (jogging):ti,ab,kw or (calisthenics):ti,ab,kw or (skating):ti,ab,kw or (Climbing):ti,ab,kw or (Pilates):ti,ab,kw or (Tai-ji):ti,ab,kw or (Tai Chi):ti,ab,kw or (wuqinxi):ti,ab,kw or (yoga):ti,ab,kw or (Five animal exercises):ti,ab,kw or (traditional chinese exercise):ti,ab,kw or (Baduanjin exercise):ti,ab,kw or (Resistance training):ti,ab,kw or (Training, Resistance):ti,ab,kw or (Strength Training):ti,ab,kw or (Training, Strength):ti,ab,kw or (Weight-Lifting Strengthening Program):ti,ab,kw or (Weight-Lifting Exercise Program):ti,ab,kw or (Blood Flow Restriction Therapy):ti,ab,kw or(BFR Therapies):ti,ab,kw or (BFRT):ti,ab,kw or (blood flow restriction):ti,ab,kw or (KAATSU):ti,ab,kw or (vascular restriction):ti,ab,kw or (Occlusion training):ti,ab,kw

#3 #1 AND #2

**Search strategy of Pubmed**

#1 (("Osteoarthritis, Knee"[Mesh]) OR (Knee Osteoarthritis[Title/Abstract]) OR (Osteoarthritides[Title/Abstract]) OR (Osteoarthrosis[Title/Abstract]) OR (Osteoarthroses[Title/Abstract]) OR ("Arthritis, Degenerative"[Title/Abstract]) OR ("Arthritides, Degenerative"[Title/Abstract]) OR ("Degenerative Arthritides"[Title/Abstract]) OR ("Degenerative Arthritis"[Title/Abstract]) OR (Arthrosis[Title/Abstract]) OR (Arthroses[Title/Abstract]) OR ("Osteoarthrosis Deformans"[Title/Abstract]))

#2 (("Exercise Therapy"[Mesh]) OR ("Exercise"[Mesh]) OR ("Motor Activity"[Mesh]) OR (Aerobics[Title/Abstract]) OR ("Physical Exercise"[Title/Abstract]) OR ("Aerobic Exercise"[Title/Abstract]) OR (walk[Title/Abstract]) OR (run[Title/Abstract]) OR (dance[Title/Abstract]) OR (swim[Title/Abstract]) OR ("aquatic exercises"[Title/Abstract]) OR (bike[Title/Abstract]) OR (bicycle[Title/Abstract]) OR (treadmill[Title/Abstract]) OR (jogging[Title/Abstract]) OR (calisthenics[Title/Abstract]) OR (skating[Title/Abstract]) OR (Climbing[Title/Abstract]) OR (Pilates[Title/Abstract]) OR ("Tai-ji"[Title/Abstract]) OR ("Tai Chi"[Title/Abstract]) OR (wuqinxi[Title/Abstract]) OR (yoga[Title/Abstract]) OR ("Five animal exercises"[Title/Abstract]) OR ("traditional chinese exercise"[Title/Abstract]) OR ("Baduanjin exercise"[Title/Abstract]) OR ("Resistance training"[Title/Abstract]) OR ("Training, Resistance"[Title/Abstract]) OR ("Strength Training"[Title/Abstract]) OR ("Training, Strength"[Title/Abstract]) OR ("Weight-Lifting Strengthening Program"[Title/Abstract]) OR ("Weight-Lifting Exercise Program"[Title/Abstract]) OR ("Blood Flow Restriction Therapy"[Title/Abstract]) OR ("BFR Therapies"[Title/Abstract]) OR (BFRT[Title/Abstract]) OR ("blood flow restriction"[Title/Abstract]) OR (KAATSU[Title/Abstract]) OR ("vascular restriction"[Title/Abstract]) OR ("Occlusion training"[Title/Abstract]))

#3 #1 AND #2

**Search strategy of Wos**

Search: TS=(("Knee Osteoarthritis" OR Osteoarthritides OR Osteoarthrosis OR Osteoarthroses OR "Arthritis, Degenerative" OR "Degenerative Arthritides" OR "Degenerative Arthritis" OR Arthrosis OR Arthroses OR "Osteoarthrosis Deformans") AND (Aerobics OR "Physical Exercise" OR "Aerobic Exercise" OR walk OR run OR dance OR swim OR "aquatic exercises" OR bike OR bicycle OR treadmill OR jogging OR calisthenics OR skating OR Climbing OR Pilates OR "Tai-ji" OR "Tai Chi" OR wuqinxi OR yoga OR "Five animal exercises" OR "traditional chinese exercise" OR "Baduanjin exercise" OR "Resistance training" OR "Training, Resistance" OR "Strength Training" OR "Training, Strength" OR "Weight-Lifting Strengthening Program" OR "Weight-Lifting Exercise Program" OR "Blood Flow Restriction Therapy" OR "BFR Therapies" OR BFRT OR "blood flow restriction" OR KAATSU OR "vascular restriction" OR "Occlusion training"))

**Search strategy of Embase**

Search: (('knee osteoarthritis'/exp) OR ('knee osteoarthritis':ti,ab,kw) OR ('osteoarthritides':ti,ab,kw) OR ('osteoarthrosis':ti,ab,kw) OR ('osteoarthroses':ti,ab,kw) OR ('arthritis, degenerative':ti,ab,kw) OR ('arthritides, degenerative':ti,ab,kw) OR ('degenerative arthritides':ti,ab,kw) OR ('degenerative arthritis':ti,ab,kw) AND (('exercise'/exp) OR ('exercise therapy'/exp) OR ('motor activity'/exp) OR (aerobics:ti,ab,kw) OR ('physical exercise':ti,ab,kw) OR ('aerobic exercise':ti,ab,kw) OR (walk:ti,ab,kw) OR (run:ti,ab,kw) OR (dance:ti,ab,kw) OR (swim:ti,ab,kw) OR ('aquatic exercises':ti,ab,kw) OR (bike:ti,ab,kw) OR (bicycle:ti,ab,kw) OR (treadmill:ti,ab,kw) OR (jogging:ti,ab,kw) OR (calisthenics:ti,ab,kw) OR (skating:ti,ab,kw) OR (climbing:ti,ab,kw) OR (pilates:ti,ab,kw) OR ('tai-ji':ti,ab,kw) OR ('tai chi':ti,ab,kw) OR (wuqinxi:ti,ab,kw) OR (yoga:ti,ab,kw) OR ('five animal exercises':ti,ab,kw) OR ('traditional chinese exercise':ti,ab,kw) OR ('baduanjin exercise':ti,ab,kw) OR ('resistance training':ti,ab,kw) OR ('training, resistance':ti,ab,kw) OR ('strength training':ti,ab,kw) OR ('training, strength':ti,ab,kw) OR ('blood flow restriction therapy':ti,ab,kw) OR ('BFR therapies':ti,ab,kw) OR ('BFRT':ti,ab,kw) OR ('blood flow restriction':ti,ab,kw) OR ('KAATSU':ti,ab,kw) OR ('vascular restriction':ti,ab,kw) OR ('occlusion training':ti,ab,kw))

**Search strategy of Ebsco**

Search: ((TI ("Knee Osteoarthritis" OR Osteoarthritides OR Osteoarthrosis OR Osteoarthroses OR "Arthritis, Degenerative" OR "Degenerative Arthritis" OR (AB ("Knee Osteoarthritis" OR Osteoarthritides OR "Degenerative Arthritis" OR Arthroses"))) AND ((TI (Aerobics OR "Physical Exercise" OR "Aerobic Exercise" OR walk OR run OR dance OR swim OR "aquatic exercises" OR bike OR bicycle OR treadmill OR jogging OR calisthenics OR skating OR Climbing OR Pilates OR "Tai-ji" OR wuqinxi OR yoga OR "Five animal exercises" OR "traditional chinese exercise" OR "Baduanjin exercise" OR "Resistance training" OR "Training, Resistance" OR "Strength Training" OR "Blood Flow Restriction Therapy" OR "BFR Therapies" OR BFRT OR "blood flow restriction" OR KAATSU OR "Occlusion training")) OR (AB (Aerobics OR "Physical Exercise" OR "Aerobic Exercise" OR walk OR run OR dance OR swim OR "aquatic exercises" OR bike OR bicycle OR treadmill OR jogging OR calisthenics OR skating OR Climbing OR Pilates OR "Tai-ji" OR wuqinxi OR yoga OR "Five animal exercises" OR "traditional chinese exercise" OR "Baduanjin exercise" OR "Resistance training" OR "Training, Resistance" OR "Training, Strength" OR "Blood Flow Restriction Therapy" OR "BFR Therapies" OR BFRT OR "blood flow restriction" OR KAATSU OR "vascular restriction" OR "Occlusion training")))

**Appendix 3 The classifications of exercise training.**

| **Type** | **Definition** |
| --- | --- |
| CON(Control) | No exercise |
|  | Educational observation |
|  | Attention control |
| MB(Mind Body exercise) | Mind-body exercise included interventions emphasizing coordinated movement, breathing regulation, postural control, and mind-body integration (e.g., Tai Chi, Yoga, Qigong, and Baduanjin). |
|  |  |
|  |  |
| ST(Strength Training) | Strength training included interventions primarily designed to improve muscle strength or endurance through external or bodyweight resistance. |
|  |  |
|  |  |
|  |  |
| AE(Aerobic Exercise) | Aerobic exercise included interventions mainly aimed at improving cardiorespiratory endurance through continuous rhythmic activity, such as walking or cycling. |
| BFRT(Blood Flow Restriction Therapy) | BFRT referred to low-load exercise performed with external blood flow restriction as a defining training feature. |
|  |  |
|  |  |
|  |  |
| MEx (Mixed Exercise) | Mixed exercise was defined as an intervention combining two or more co-primary exercise components when no single dominant component could be identified. |
|  |  |
|  |  |
|  |  |

When an intervention contained multiple components but one training component was clearly predominant in structure or intended effect, the study was classified according to that dominant component. Two reviewers independently performed the classification, and disagreements were resolved through discussion.

**Appendix 4** **Risk of bias assessment.**

**Appendix 5 Contributions of direct and indirect comparisons to NMA and the number of studies for each direct comparison of pain, function, stiffness, and muscle strength.**

Note: A-CON, B-MB, C-ST, D-AE, E-BFRT, F-MEx

Appendix 5-1 Pain

Appendix 5-2 Function

Appendix 5-3 Stiffness

Appendix 5-4 Muscle Strength

**Appendix 6. Inconsistency of outcomes tested by loop-specific heterogeneity estimates, inconsistency model and node splitting analysis.**

Note: A-CON, B-MB, C-ST, D-AE, E-BFRT, F-MEx MS：Muscle Strength

| loop-specific heterogeneity estimates | | | | | | |
| --- | --- | --- | --- | --- | --- | --- |
| Loop | IF | seIF | z_value | p_value | CI_95 | Loop_Heterog_tau2 |
|  |  |  | **Pain** |  |  |  |
| CON-MB-MEx | 0.848 | 1.169 | 0.725 | 0.468 | (0.00,3.14) | 0.722 |
| CON-ST-BFRT | 0.503 | 0.655 | 0.769 | 0.442 | (0.00,1.79) | 0.19 |
| MB-ST-MEx | 0.482 | 1.005 | 0.48 | 0.631 | (0.00,2.45) | 0.797 |
| CON-ST-MEx | 0.385 | 0.782 | 0.492 | 0.623 | (0.00,1.92) | 0.3 |
| MB-ST-AE | 0.375 | 0.638 | 0.587 | 0.557 | (0.00,1.63) | 0.125 |
| CON-ST-AE | 0.188 | 0.531 | 0.354 | 0.723 | (0.00,1.23) | 0.322 |
| CON-MB-AE | 0.153 | 1.001 | 0.152 | 0.879 | (0.00,2.12) | 0.522 |
| CON-MB-ST | 0.014 | 0.374 | 0.036 | 0.971 | (0.00,0.75) | 0.303 |
|  |  |  | **Function** |  |  |  |
| CON-ST-MEx | 1.475 | 1.14 | 1.293 | 0.196 | (0.00,3.71) | 0.553 |
| CON-MB-MEx | 0.998 | 0.849 | 1.176 | 0.24 | (0.00,2.66) | 0.347 |
| MB-ST-MEx | 0.693 | 0.968 | 0.715 | 0.474 | (0.00,2.59) | 0.473 |
| CON-ST-AE | 0.322 | 0.708 | 0.454 | 0.65 | (0.00,1.71) | 0.526 |
| CON-MB-ST | 0.27 | 0.417 | 0.648 | 0.517 | (0.00,1.09) | 0.335 |
|  |  |  | **Stiffness** |  |  |  |
| CON-MB-ST | 0.899 | 0.615 | 1.462 | 0.144 | (0.00,2.10) | 0.375 |
| CON-ST-AE | 0.781 | 1.124 | 0.695 | 0.487 | (0.00,2.98) | 0.58 |
| CON-MB-MEx | 0.37 | 1.119 | 0.331 | 0.741 | (0.00,2.56) | 0.493 |
|  |  |  | **MS** |  |  |  |
| CON-MB-AE | 1.515 | 0.653 | 2.321 | 0.02 | (0.24,2.79) | 0.124 |
| CON-MB-ST | 1.018 | 0.604 | 1.685 | 0.092 | (0.00,2.20) | 0.218 |
| CON-ST-BFRT | 0.584 | 0.882 | 0.662 | 0.508 | (0.00,2.31) | 0.235 |
| MB-ST-AE | 0.584 | 0.415 | 1.406 | 0.16 | (0.00,1.40) | 0 |
| CON-ST-AE | 0.188 | 0.462 | 0.408 | 0.683 | (0.00,1.09) | 0.159 |

| Inconsistency model | | | | |
| --- | --- | --- | --- | --- |
|  | **Pain** | **Function** | **Stiffness** | **MS** |
| chi2( 12) | 7.2 | 7.03 | 7.96 | 6.97 |
| Prob > chi2 | 0.8444 | 0.533 | 0.2411 | 0.324 |

| Node splitting analysis | | | | | | |  |
| --- | --- | --- | --- | --- | --- | --- | --- |
| Side | Direct |  | Indirect |  | Difference |  |  |
|  | Coef. | Std. Err. | Coef. | Std. Err. | Coef. | Std. Err. | P>z |
|  |  |  | **Pain** |  |  |  |  |
| A B | -1.176 | 0.197 | -0.960 | 0.386 | -0.215 | 0.433 | 0.619 |
| A C | -0.701 | 0.198 | -0.635 | 0.329 | -0.067 | 0.384 | 0.862 |
| A D | -0.852 | 0.215 | -1.029 | 0.579 | 0.178 | 0.617 | 0.774 |
| A E | -0.507 | 0.782 | -1.058 | 0.433 | 0.551 | 0.894 | 0.538 |
| A F | -0.622 | 0.756 | -1.162 | 0.481 | 0.541 | 0.898 | 0.547 |
| B C | 0.423 | 0.294 | 0.473 | 0.290 | -0.050 | 0.413 | 0.904 |
| B D | 0.481 | 0.775 | 0.231 | 0.272 | 0.250 | 0.821 | 0.761 |
| B F | -0.288 | 0.518 | 0.733 | 0.630 | -1.022 | 0.816 | 0.210 |
| C D | -0.260 | 0.433 | -0.156 | 0.298 | -0.105 | 0.526 | 0.842 |
| C E | -0.354 | 0.397 | 0.197 | 0.801 | -0.551 | 0.894 | 0.538 |
| C F | -0.320 | 0.527 | -0.326 | 0.622 | 0.006 | 0.815 | 0.994 |
|  |  |  | **Function** |  |  |  |  |
| A B | -1.314 | 0.203 | -1.145 | 0.462 | -0.169 | 0.505 | 0.737 |
| A C | -0.867 | 0.226 | -1.054 | 0.405 | 0.187 | 0.464 | 0.687 |
| A D * | -0.785 | 0.258 | -1.953 | 0.948 | 1.169 | 0.982 | 0.234 |
| A F | -0.553 | 0.702 | -1.987 | 0.560 | 1.435 | 0.901 | 0.111 |
| B C | 0.230 | 0.330 | 0.510 | 0.319 | -0.281 | 0.459 | 0.541 |
| B F * | -0.233 | 0.505 | 0.186 | 0.969 | -0.419 | 1.092 | 0.701 |
| C D | -0.071 | 0.500 | 0.109 | 0.375 | -0.180 | 0.625 | 0.774 |
| C E * | -0.160 | 0.534 | 1.660 | 141.386 | -1.820 | 141.386 | 0.990 |
| C F | -1.166 | 0.693 | -0.037 | 0.599 | -1.130 | 0.915 | 0.217 |
|  |  |  | **Stiffness** |  |  |  |  |
| A B * | -1.214 | 0.239 | -0.536 | 0.716 | -0.678 | 0.755 | 0.369 |
| A C | -0.633 | 0.418 | -1.097 | 0.566 | 0.464 | 0.704 | 0.510 |
| A D * | -0.531 | 0.334 | -2.717 | 1.537 | 2.186 | 1.572 | 0.164 |
| A F * | -1.007 | 0.828 | -0.119 | 1.503 | -0.888 | 1.728 | 0.607 |
| B C | -0.226 | 0.456 | 0.990 | 0.476 | -1.216 | 0.659 | 0.065 |
| B F * | 0.549 | 0.818 | -0.339 | 1.519 | 0.888 | 1.728 | 0.607 |
| C D | -0.663 | 0.786 | 0.527 | 0.521 | -1.190 | 0.943 | 0.207 |
| C E * | -0.357 | 0.597 | 1.232 | 141.467 | -1.589 | 141.468 | 0.991 |
|  |  |  | **MS** |  |  |  |  |
| A B | -0.106 | 0.365 | 0.991 | 0.322 | -1.097 | 0.488 | 0.025 |
| A C | 0.852 | 0.190 | 0.586 | 0.364 | 0.266 | 0.411 | 0.517 |
| A D | 0.799 | 0.232 | 0.074 | 0.431 | 0.726 | 0.490 | 0.139 |
| A E | 1.068 | 0.548 | 0.388 | 0.566 | 0.680 | 0.788 | 0.388 |
| B C | -0.033 | 0.331 | 0.766 | 0.408 | -0.800 | 0.526 | 0.129 |
| B D | -0.612 | 0.496 | 0.503 | 0.357 | -1.115 | 0.611 | 0.068 |
| C D | -0.180 | 0.354 | -0.149 | 0.346 | -0.031 | 0.494 | 0.950 |
| C E | -0.375 | 0.540 | 0.305 | 0.574 | -0.680 | 0.788 | 0.388 |

* All the evidence about these contrasts comes from the trials which directly compare them.

**Appendix 7 Forest plots of eligible comparisons of pain,function,stiffness,muscle strength.**

Appendix 7-1 Pain

Appendix 7-2 Function

Appendix 7-3 Stiffness

Appendix 7-4 Muscle Strength

**Appendix 8 Funnel plot**

Pain	Function

Stiffness	Muscle strength

**Appendix 9 Restricted analyses of primary outcomes according to age and intervention duration**

**Restricted analysis of pain**

| **Age** | MB |  |  |  |  |  |
| --- | --- | --- | --- | --- | --- | --- |
|  | -0.35 (-0.82,0.12) | ST |  |  |  |  |
|  | -0.23 (-0.82,0.37) | 0.12 (-0.46,0.71) | AE |  |  |  |
|  | -0.11 (-0.92,0.70) | 0.24 (-0.45,0.94) | 0.12 (-0.76,1.00) | BFRT |  |  |
|  | -0.07 (-0.87,0.73) | 0.28 (-0.50,1.07) | 0.16 (-0.76,1.08) | 0.04 (-0.99,1.08) | MEx |  |
|  | **-1.05 (-1.42,-0.67)** | **-0.69 (-1.08,-0.31)** | **-0.82 (-1.29,-0.35)** | **-0.94 (-1.69,-0.18)** | **-0.98 (-1.77,-0.18)** | CON |
| **Duration** | MB |  |  |  |  |  |
|  | -0.32 (-0.75,0.12) | ST |  |  |  |  |
|  | -0.27 (-0.92,0.38) | 0.04 (-0.60,0.69) | AE |  |  |  |
|  | 0.05 (-0.94,1.04) | 0.36 (-0.53,1.26) | 0.32 (-0.78,1.42) | BFRT |  |  |
|  | -0.06 (-0.85,0.74) | 0.26 (-0.52,1.05) | 0.22 (-0.74,1.18) | -0.10 (-1.29,1.09) | MEx |  |
|  | **-1.07 (-1.43,-0.72)** | **-0.76 (-1.13,-0.39)** | **-0.80 (-1.36,-0.25)** | **-1.12 (-2.09,-0.16)** | **-1.02 (-1.81,-0.22)** | CON |

**Restricted analysis of Function**

| **Age** | MB |  |  |  |  |  |
| --- | --- | --- | --- | --- | --- | --- |
|  | -0.34 (-0.87,0.20) | ST |  |  |  |  |
|  | -0.43 (-1.12,0.26) | -0.09 (-0.80,0.62) | AE |  |  |  |
|  | -0.18 (-1.41,1.06) | 0.16 (-0.95,1.27) | 0.25 (-1.06,1.57) | BFRT |  |  |
|  | 0.14 (-0.79,1.06) | 0.47 (-0.50,1.45) | 0.57 (-0.53,1.66) | 0.31 (-1.16,1.79) | MEx |  |
|  | **-1.35 (-1.75,-0.94)** | **-1.01 (-1.49,-0.54)** | **-0.92 (-1.49,-0.35)** | **-1.17 (-2.38,0.04)** | **-1.48 (-2.43,-0.54)** | CON |
| **Duration** | MB |  |  |  |  |  |
|  | -0.25 (-0.71,0.22) | ST |  |  |  |  |
|  | -0.38 (-1.11,0.34) | -0.14 (-0.86,0.59) | AE |  |  |  |
|  | -0.35 (-1.91,1.20) | -0.11 (-1.59,1.37) | 0.03 (-1.62,1.68) | BFRT |  |  |
|  | 0.18 (-0.69,1.06) | 0.43 (-0.48,1.34) | 0.57 (-0.51,1.65) | 0.54 (-1.20,2.28) | MEx |  |
|  | **-1.25 (-1.62,-0.88)** | **-1.00 (-1.43,-0.58)** | **-0.87 (-1.50,-0.24)** | **-0.90 (-2.44,0.64)** | **-1.44 (-2.32,-0.55)** | CON |

**Restricted analysis of Stiffness**

| **Age** | MB |  |  |  |  |  |
| --- | --- | --- | --- | --- | --- | --- |
|  | -0.40 (-1.19,0.39) | ST |  |  |  |  |
|  | -0.55 (-1.38,0.28) | -0.15 (-1.08,0.78) | AE |  |  |  |
|  | -0.04 (-1.50,1.41) | 0.36 (-0.87,1.58) | 0.51 (-1.03,2.04) | BFRT |  |  |
|  | -0.37 (-1.82,1.09) | 0.03 (-1.57,1.64) | 0.18 (-1.42,1.79) | -0.32 (-2.34,1.69) | MEx |  |
|  | **-1.18 (-1.67,-0.69)** | **-0.78 (-1.50,-0.06)** | -0.63 (-1.32,0.05) | -1.14 (-2.56,0.28) | -0.82 (-2.27,0.64) | CON |
| **Duration** | MB |  |  |  |  |  |
|  | -0.13 (-0.91,0.65) | ST |  |  |  |  |
|  | -0.58 (-1.58,0.43) | -0.44 (-1.62,0.73) | AE |  |  |  |
|  | -0.35 (-1.83,1.13) | 0.19 (-1.56,1.94) | 0.63 (-1.47,2.74) | BFRT |  |  |
|  | -0.37 (-1.82,1.09) | -0.22 (-1.86,1.42) | 0.23 (-1.50,1.95) | -0.40 (-2.80,1.99) | MEx |  |
|  | **-1.15 (-1.63,-0.66)** | **-1.01 (-1.80,-0.23)** | -0.57 (-1.45,0.31) | -1.20 (-3.12,0.71) | -0.80 (-2.28,0.69) | CON |

**Restricted analysis of Muscle strength**

| **Age** | MB |  |  |  |  |
| --- | --- | --- | --- | --- | --- |
|  | -0.67 (-1.59,0.24) | ST |  |  |  |
|  | -0.34 (-1.85,1.17) | 0.33 (-0.96,1.63) | AE |  |  |
|  | -0.64 (-1.92,0.63) | 0.03 (-0.92,0.98) | -0.31 (-1.83,1.22) | BFRT |  |
|  | 0.07 (-0.86,1.00) | **0.74 (0.24,1.24)** | **0.41 (-0.78,1.60)** | 0.71 (-0.24,1.67) | **C**ON |
| **Duration** | MB |  |  |  |  |
|  | -0.56 (-1.19,0.08) | ST |  |  |  |
|  | -0.64 (-1.44,0.17) | -0.08 (-0.68,0.52) | AE |  |  |
|  | -0.50 (-1.47,0.47) | 0.06 (-0.73,0.85) | 0.14 (-0.80,1.08) | BFRT |  |
|  | 0.24 (-0.37,0.86) | **0.80 (0.44,1.16)** | **0.88 (0.34,1.42)** | 0.74 (-0.05,1.53) | CON |

Note: Values are presented as effect sizes with 95% confidence intervals (CIs) for pairwise comparisons between the row and column interventions within each restricted analysis. Age refers to analyses restricted to studies involving participants aged 50–60 years, whereas Duration refers to analyses conducted after excluding short-term interventions (4–7 weeks). For pain, function, and stiffness, values < 0 favor the row intervention, whereas values > 0 favor the column intervention. For muscle strength, values > 0 favor the row intervention, whereas values < 0 favor the column intervention. Bold values indicate statistically significant differences between interventions.

**Appendix 10** SUCRA ranking for the restricted analyses by age and intervention duration

| **Pain** | **Age** |  |  | **Duration** |  |
| --- | --- | --- | --- | --- | --- |
|  | Treatment | SUCRA |  | Treatment | SUCRA |
|  | MB | 77.2 |  | MB | 74.4 |
|  | MEx | 67.5 |  | BFRT | 71.8 |
|  | BFRT | 64.1 |  | MEx | 66 |
|  | AE | 52.8 |  | AE | 47.7 |
|  | ST | 38 |  | ST | 39.8 |
|  | CON | 0.3 |  | CON | 0.4 |
| **Function** | **Age** |  |  | **Duration** |  |
|  | Treatment | SUCRA |  | Treatment | SUCRA |
|  | MEx | 78.9 |  | MEx | 81.2 |
|  | MB | 75.7 |  | MB | 74.2 |
|  | BFRT | 59 |  | ST | 51.1 |
|  | ST | 45.2 |  | BFRT | 48.1 |
|  | AE | 40.6 |  | AE | 42.7 |
|  | CON | 0.6 |  | CON | 2.6 |
| **Stiffness** | **Age** |  |  | **Duration** |  |
|  | Treatment | SUCRA |  | Treatment | SUCRA |
|  | MB | 78.9 |  | MB | 73.1 |
|  | BFRT | 70 |  | BFRT | 66.6 |
|  | MEx | 53.7 |  | ST | 63.2 |
|  | ST | 50.5 |  | MEx | 51.3 |
|  | AE | 42.2 |  | AE | 38.3 |
|  | CON | 4.8 |  | CON | 7.5 |
| **Muscle strength** | **Age** |  |  | **Duration** |  |
|  | Treatment | SUCRA |  | Treatment | SUCRA |
|  | ST | 78.6 |  | AE | 83.7 |
|  | BFRT | 72.5 |  | ST | 75.5 |
|  | AE | 51.4 |  | BFRT | 46.3 |
|  | MB | 28.2 |  | MB | 31.7 |
|  | CON | 19.3 |  | CON | 12.9 |

**Appendix 11 GRADE for the outcomes.**

1. Summary of study limitations of the included studies.

The colours of the line indicate the summative ROB assessment of each comparison based on ROB assessmentof each included studies (low ROB comparison [green], moderate ROB comparison [yellow] and high ROB comparison [red]).

Note: A-CON, B-MB, C-ST, D-AE, E-BFRT, F-MEx

Pain	Function

Stiffness	Muscle Strength

2. Contribution of ROB comparisons to each network estimate.

Based on the above assessment of ROB for each comparison and the contributions of direct and indirect comparisons to all network estimates, the following bar graphs show the percentage of low, moderate and high ROB contributions for each network estimate.

Pain

`

Function

Stiffness

Muscle strength

**3. Table of reasons for downgrading**

Based on all the above information, we GRADEd each network estimate according to the following criteria.

(1) Study limitations

Low, unclear, and high risk of bias for each direct comparison were assigned weights of 0, −1, and −2, respectively. The risk value for each network estimate was calculated according to the contribution of risk-of-bias comparisons to that network estimate. Evidence was downgraded by one level when the risk value was between −1.5 and −0.6, indicating serious study limitations, and by two levels when the risk value was less than −1.6, indicating very serious study limitations. These are reported as “Study limitations (serious)” and “Study limitations (very serious)”, respectively.

(2) Indirectness

In the network plot, singly-connected nodes were slightly downgraded in terms of indirectness because assessing transitivity for these nodes is unclear. To put it simply, if the results of two comparisons are obtained solely through indirect comparisons rather than direct ones, the reliability of the outcome will be downgraded by one level.

(3) Inconsistency

In this study, we evaluated heterogeneity and inconsistency. Heterogeneity for each direct comparison was assessed using the prediction intervals presented in the pairwise forest plots (Appendix 7). When inconsistency was observed, it was categorized as high heterogeneity (labeled as “heterogeneous”). Inconsistency between direct and indirect evidence was evaluated using loop-specific inconsistency estimates and node-splitting analyses (Appendix 6) and was labeled as “Inconsistency.”

(4) Imprecision

Imprecision was evaluated based on sample size; a sample size below 400 resulted in a one-level downgrade. The total sample size in this study was over 400, so this project was not downgraded. The Imprecision of the ranking results is evaluated according to the gap between the ranking values. If the differences between treatment ranking values were small, indicating poor ranking stability, the evidence was downgraded by one level.

(5) Publication bias

We evaluated the potential publication bias by checking the symmetry of pairwise comparisons in the adjusted funnel plot. Evidence was downgraded when publication bias was considered present. The GRADE assessments for the outcomes are presented below.

**Pain**

| Comparison | Nature of the evidence | Confidence | Downgrading due to |
| --- | --- | --- | --- |
| CON vs MB | Mixed | Very low | Study limitations (serious),  Heterogeneity, Publication bias |
| CON vs ST | Mixed | Low | Study limitations (serious), Heterogeneity |
| CON vs AE | Mixed | Very low | Study limitations (serious),  Heterogeneity, Publication bias |
| CON vs BFRT | Mixed | Low | Study limitations (serious), Heterogeneity |
| CON vs MEx | Mixed | Very low | Study limitations (very serious), Heterogeneity |
| MB vs ST | Mixed | Low | Study limitations (serious), Heterogeneity |
| MB vs AE | Mixed | Moderate | Study limitations (serious) |
| MB vs MEx | Mixed | Low | Study limitations (very serious) |
| ST vs AE | Mixed | Moderate | Study limitations (serious) |
| ST vs BFRT | Mixed | High |  |
| ST vs MEx | Mixed | Low | Study limitations (very serious) |
| MB vs BFRT | Indirect | Low | Study limitations (serious), Indirectness |
| AE vs BFRT | Indirect | Low | Study limitations (serious), Indirectness |
| AE vs MEx | Indirect | Very low | Study limitations (very serious), Indirectness |
| BFRT vs MEx | Indirect | Low | Study limitations (serious), Indirectness |
| Ranking of treatments |  | Moderate | Study limitations (serious) |

**Function**

| Comparison | Nature of  the evidence | Confidence | Downgrading due to |
| --- | --- | --- | --- |
| CON vs MB | Mixed | Very low | Study limitations (serious),  Heterogeneity, Publication bias |
| CON vs ST | Mixed | Very low | Study limitations (serious),  Heterogeneity, Publication bias |
| CON vs AE | Mixed | Very low | Study limitations (serious),  Heterogeneity, Publication bias |
| CON vs MEx | Mixed | Low | Study limitations (serious), Heterogeneity |
| MB vs ST | Mixed | Moderate | Study limitations (serious) |
| MB vs MEx | Mixed | Low | Study limitations (serious), Publication bias |
| ST vs AE | Mixed | Low | Study limitations (very serious) |
| ST vs BFRT | Mixed | Low | Study limitations (very serious) |
| ST vs MEx | Mixed | Moderate | Study limitations (serious) |
| CON vs BFRT | Indirect | Low | Study limitations (serious), Indirectness |
| MB vs AE | Indirect | Low | Study limitations (serious), Indirectness |
| MB vs BFRT | Indirect | Low | Study limitations (serious), Indirectness |
| AE vs BFRT | Indirect | Very low | Study limitations (very serious), Indirectness |
| AE vs MEx | Indirect | Very low | Study limitations (very serious), Indirectness |
| BFRT vs MEx | Indirect | Very low | Study limitations (very serious), Indirectness |
| Ranking of treatments |  | Moderate | Study limitations (serious) |

**Stiffness**

| Comparison | Nature of the evidence | Confidence | Downgrading due to |
| --- | --- | --- | --- |
| CON vs MB | Mixed | Very low | Study limitations (serious),  Heterogeneity, Publication bias |
| CON vs ST | Mixed | Very low | Study limitations (serious),  Heterogeneity, Publication bias |
| CON vs AE | Mixed | Moderate | Study limitations (serious) |
| CON vs MEx | Mixed | Low | Study limitations (very serious) |
| MB vs ST | Mixed | Low | Study limitations (serious), Publication bias |
| MB vs MEx | Mixed | Very low | Study limitations (very serious), Publication bias |
| ST vs AE | Mixed | Low | Study limitations (very serious) |
| ST vs BFRT | Mixed | Moderate | Study limitations (serious) |
| CON vs BFRT | Indirect | Low | Study limitations (serious), Indirectness |
| MB vs AE | Indirect | Low | Study limitations (serious), Indirectness |
| MB vs BFRT | Indirect | Low | Study limitations (serious), Indirectness |
| ST vs MEx | Indirect | Low | Study limitations (serious), Indirectness |
| AE vs BFRT | Indirect | Low | Study limitations (serious), Indirectness |
| AE vs MEx | Indirect | Very low | Study limitations (very serious), Indirectness |
| BFRT vs MEx | Indirect | Low | Study limitations (serious), Indirectness |
| Ranking of treatments |  | Moderate | Study limitations (serious) |

**Muscle strength**

| Comparison | Nature of the evidence | Confidence | Downgrading due to |
| --- | --- | --- | --- |
| CON vs MB | Mixed | Low | Study limitations (serious), Inconsistency |
| CON vs ST | Mixed | Very low | Study limitations (serious),  Heterogeneity, Publication bias |
| CON vs AE | Mixed | Low | Study limitations (serious), Heterogeneity |
| CON vs BFRT | Mixed | Moderate | Study limitations (serious) |
| MB vs ST | Mixed | Low | Study limitations (serious), Publication bias |
| MB vs AE | Mixed | Very low | Study limitations (very serious), Publication bias |
| ST vs AE | Mixed | Low | Study limitations (very serious) |
| ST vs BFRT | Mixed | Moderate | Study limitations (serious) |
| MB vs BFRT | Indirect | Low | Study limitations (serious), Indirectness |
| AE vs BFRT | Indirect | Low | Study limitations (serious), Indirectness |
| Ranking of treatments |  | Moderate | Study limitations (serious) |
